# Pathobiological investigation of naturally infected canine rabies cases from Sri Lanka

**DOI:** 10.1186/s12917-017-1024-5

**Published:** 2017-04-12

**Authors:** S. Beck, P. Gunawardena, D. L. Horton, D. J. Hicks, D. A. Marston, A. Ortiz-Pelaez, A. R. Fooks, A. Núñez

**Affiliations:** 1 Pathology Department, Animal and Plant Health Agency, Weybridge, UK; 2 Department of Veterinary Pathobiology, University of Peradeniya, Peradeniya, Sri Lanka; 3 Animal and Plant Health Agency, Weybridge, UK; 4 Wildlife Zoonoses and Vector Borne Diseases Research Group, Animal and Plant Health Agency, Weybridge, UK

**Keywords:** Rabies canine histopathology immunohistochemistry hemi-nested reverse transcription polymerase chain reaction

## Abstract

**Background:**

The recommended screening of rabies in ‘suspect’ animal cases involves testing fresh brain tissue. The preservation of fresh tissue however can be difficult under field conditions and formalin fixation provides a simple alternative that may allow a confirmatory diagnosis. The occurrence and location of histopathological changes and immunohistochemical (IHC) labelling for rabies in formalin fixed paraffin embedded (FFPE) canine brain is described in samples from 57 rabies suspect cases from Sri-Lanka. The presence of Negri bodies and immunohistochemical detection of rabies virus antigen were evaluated in the cortex, hippocampus, cerebellum and brainstem. The effect of autolysis and artefactual degeneration of the tissue was also assessed.

**Results:**

Rabies was confirmed in 53 of 57 (93%) cases by IHC. IHC labelling was statistically more abundant in the brainstem. Negri bodies were observed in 32 of 53 (60.4%) of the positive cases. Although tissue degradation had no effect on IHC diagnosis, it was associated with an inability to detect Negri bodies. In 13 cases, a confirmatory Polymerase chain reaction (PCR) testing for rabies virus RNA was undertaken by extracting RNA from fresh frozen tissue, and also attempted using FFPE samples. PCR detection using fresh frozen samples was in agreement with the IHC results. The PCR method from FFPE tissues was suitable for control material but unsuccessful in our field cases.

**Conclusions:**

Histopathological examination of the brain is essential to define the differential diagnoses of behaviour modifying conditions in rabies virus negative cases, but it is unreliable as the sole method for rabies diagnosis, particularly where artefactual change has occurred. Formalin fixation and paraffin embedding does not prevent detection of rabies virus via IHC labelling even where artefactual degeneration has occurred. This could represent a pragmatic secondary assay for rabies diagnosis in the field because formalin fixation can prevent sample degeneration. The brain stem was shown to be the site with most viral immunoreactivity; supporting recommended sampling protocols in favour of improved necropsy safety in the field. PCR testing of formalin fixed tissue may be successful in certain circumstances as an alternative test.

## Background

Rabies is a highly neurotropic virus which is thought to have the potential to infect most mammalian species [10]. There is an almost universal case fatality rate (approaching 100%) in humans, for whom the predominant source of exposure leading to death is dog bites [14, 27]. Rabies continues to be a significant threat to human health in developing countries, with most human cases occurring in Africa and Asia [11]. There is also a need for effective rabies surveillance and diagnosis in Europe where there is a low potential risk of transmission to humans from the 7064 domestic animal and wildlife cases reported in 2013 (WHO Rabies bulletin) [20]. Even countries that are officially free of rabies such as the UK report rare, imported (quarantined) cases [21]. The ability to diagnose rabies virus in domestic animals and wildlife is the basis of most national reporting programs.

Canine rabies cannot be diagnosed by clinical presentation alone, which is often varied and may include non-specific signs such as pyrexia, altered neurological function, ataxia and paralysis prior to death [29]. Seroconversion is not a useful antemortem test because it occurs late in the disease process and there are no specific gross lesions at post-mortem [36]. Confirmation of rabies infection requires laboratory testing of brain tissue. The standard methods for the diagnosis of rabies virus in animals are recommended by the OIE (World Health Organisation for Animal Health) [31, 36]. Histopathologic examination of infected brain tissue can be used to directly identify Negri bodies in neuronal cytoplasm, which is considered pathognomonic for rabies virus infection [23]. Negri bodies are observed concurrently with perivascular, non-suppurative inflammation, glial proliferation, neuronal degeneration and necrosis. The reduced sensitivity in comparison to immunological and molecular tests has resulted in this technique no longer being recommended for primary diagnosis by the WHO or OIE [12, 36].

Many new diagnostic techniques are available for rabies diagnosis [9, 12] but the recommended (‘gold-standard’) OIE-prescribed diagnostic assay for statutory diagnosis of rabies virus is the fluorescent antibody test (FAT). This test is a direct technique undertaken on impression smears of fresh brain tissue labelling viral nucleocapsid protein [3, 15, 26]. Obtaining suitable fresh samples without a cold chain and performing testing that requires specialised local equipment remains a challenge [25, 36]. Tissue fixation provides a cheap and simple solution, by preserving samples taken in remote areas prior to laboratory analysis and also allows safe inactivation of the test material. The FAT can also be undertaken on glycerol preserved and formalin fixed tissue, with additional washing or proteolytic enzyme steps respectively, however this is less reliable then using fresh samples [36]. Immunohistochemical (IHC) methods on fixed tissues offer an alternative or additional confirmatory method to the FAT.

Heminested reverse transcription polymerase chain reaction (HnRT-PCR) and real time, Taqman reverse transcription polymerase chain reaction (Taqman RT-PCR) assays have been described [16, 17, 34]. These methods, have been reported to give 100% specificity and sensitivity [2] and Taqman RT-PCR is considered capable of detecting divergent, novel Lyssaviruses [16, 24]. PCR techniques are important confirmatory tests, often used in tandem with FAT [21], which may also be used when brain tissue is severely autolysed [8, 25]. At present PCR is not recommended for routine diagnosis by the WHO/OIE because of the requirement for exacting standards of quality control, but it has a crucial role in epidemiological analysis of virus type [13, 34, 36]. PCR is usually undertaken using fresh tissue, although HnRT-PCR has previously been unsuccessfully attempted using formalin fixed (non-paraffin embedded) tissue in a single case study [7]. PCR using viral RNA extracted from FFPE tissue would be a practical tool for rabies diagnosis where this is the only tissue available or further diagnostic confirmation is required.

A final consideration in rabies diagnosis is that viral antigen is not equally distributed throughout the brain tissue of rabies positive animals. A large case series involving multiple naturally infected species from South Africa, was tested by FAT. The brainstem was most often antigen positive in canine cases, while the hippocampus contained the largest deposits of antigen when positive [5]. Two small (10 naturally infected canids) case series suggested that viral antigen distribution is greater in the brainstem, compared to other supratentorial structures [30, 32]. Another small case series (3 naturally infected canids) suggested that the hippocampus contains the most intense signal [31]. The general anatomic location of viral antigen has also been assessed by IHC labelling in 21 rabies positive canine cases, in which the cerebellum and hippocampus most often contained positive neurones [1]. It is unknown if this variation is due to study design, stage of infection, strain differences or another factor. In murine models, infected with rabies virus or European bat lyssavirus type 1 and 2, IHC labelling revealed variation in viral antigen distribution, with the brainstem most often being positive [18, 19]. Street and laboratory (“fixed”) rabies virus have been compared using a murine model, also with variation in antigen distribution and inflammation [33]. Sample site selection is therefore crucial. The current OIE recommendation is to collect the whole brain in a necropsy room and sample hippocampus, thalamus, cerebral cortex and medulla oblongata: a pool of tissues including the brain stem is advocated for FAT sampling [5, 36]. Opening the cranium carries obvious risk for the operator and for this reason ‘straw’ techniques have been described for sampling in the field [36].

## Methods

The aim of this study is to assess in a large series of naturally infected canine rabies cases the general anatomical location of classic histopathological changes and rabies viral antigen labelling using FFPE tissue, with an analysis of the effect of artefact on detecting these features. A subset of cases were tested for rabies viral RNA by Taqman RT-PCR and HnRT-PCR using both fresh and FFPE tissue.

### Case collection

FFPE brain tissue was available from fifty seven canids submitted to the University of Peradeniya, Sri Lanka between the years of 2007-2011 (Table 1). The time for which this material was fixed prior to processing is unknown. The cases were suspected to be infected with rabies virus because of observed neurologic changes but no further clinical information was provided. The areas of the brain available for analysis varied between cases; fresh frozen tissue was available from thirteen cases.

**Table 1:** Summary of results for individual animals

Case	Cerebrum			Hippocampus			Cerebellum			Brainstem			Pooled Fresh PCR
	NB^b^	IHC^c^	Path^d^	NB	IHC	Path	NB	IHC	Path	NB	IHC	Path	
1	+	++	1/2/3/2	N/a^a^	N/a	N/a	N/a	N/a	N/a	N/a	N/a	N/a	N/a
2	−	+	1/3/2/2	N/a	N/a	N/a	N/a	N/a	N/a	N/a	N/a	N/a	N/a
3	N/a	N/a	N/a	N/a	N/a	N/a	−	+	2/0/1/2	N/a	N/a	N/a	N/a
4	+	++	0/3/2/2	+	++	0/3/2/2	+	+++	1/2/1/0	−	++	0/3/2/2	N/a
5	−	+	1/2/2/1	−	++	1/2/2/1	−	+	2/1/1/2	N/a	N/a	N/a	N/a
6	−	−	0/2/2/2	−	−	0/2/2/1	−	−	0/0/0/2	−	−	0/1/1/1	−
7	N/a	N/a	N/a	−	+	1/2/1/2	N/a	N/a	N/a	−	+++	1/2/1/2	N/a
8	−	−	0/1/1/2	N/a	N/a	N/a	−	−	0/2/2/0	−	−	0/1/1/2	N/a
9	−	−	0/1/1/2	N/a	N/a	N/a	N/a	N/a	N/a	−	+	2/2/2/1	+
10	N/a	N/a	N/a	−	++	0/2/1/1	−	++	0/1/0/1	+	+++	1/2/1/2	N/a
11	N/a	N/a	N/a	N/a	N/a	N/a	N/a	N/a	N/a	−	+++	1/2/2/1	+
12	N/a	N/a	N/a	+	+++	1/2/2/2	N/a	N/a	N/a	N/a	N/a	N/a	N/a
13	−	++	2/1/1/2	N/a	N/a	N/a	N/a	N/a	N/a	N/a	N/a	N/a	+
14	N/a	N/a	N/a	−	−	1/2/2/0	−	++	1/1/1/1	−	++	2/2/2/1	N/a
15	−	+++	1/1/1/2	N/a	N/a	N/a	+	+++	1/2/1/2	+	+++	1/1/1/1	N/a
16	−	+	0/2/1/2	−	++	2/3/2/2	−	+	2/2/1/1	−	++	2/3/3/0	N/a
17	−	++	0/2/1/1	−	++	0/2/1/1	−	++	0/1/1/1	−	+++	2/3/2/1	N/a
18	−	+++	1/2/2/0	N/a	N/a	N/a	+	++	1/1/1/1	−	+++	2/2/2/0	+
19	−	+++	0/2/1/1	−	++	0/1/2/0	N/a	N/a	N/a	N/a	N/a	N/a	+
20	−	+	0/1/1/1	N/a	N/a	N/a	N/a	N/a	N/a	N/a	N/a	N/a	+
21	+	++	1/2/3/1	+	+++	0/2/1/0	N/a	N/a	N/a	+	+++	0/2/2/0	+
22	N/a	N/a	N/a	N/a	N/a	N/a	N/a	N/a	N/a	+	+++	2/2/2/1	N/a
23	−	−	2/1/2/2	+	++	2/2/2/2	−	−	0/1/1/2	+	++	2/2/2/2	N/a
24	+	++	2/2/3/1	N/a	N/a	N/a	N/a	N/a	N/a	N/a	N/a	N/a	N/a
25	−	+++	2/2/1/1	+	+++	2/2/2/1	−	+++	3/1/1/0	−	+++	2/2/2/1	+
26	+	++	3/2/1/3	N/a	N/a	N/a	N/a	N/a	N/a	N/a	N/a	N/a	+
27	N/a	N/a	N/a	+	+++	0/3/3/0	+	+++	0/3/2/0	−	++	1/3/3/0	+
28	−	++	3/2/2/2	N/a	N/a	N/a	N/a	N/a	N/a	N/a	N/a	N/a	N/a
29	N/a	N/a	N/a	+	+++	1/3/2/2	N/a	N/a	N/a	+	+++	1/3/2/2	N/a
30	+	+++	3/3/2/0	N/a	N/a	N/a	N/a	N/a	N/a	N/a	N/a	NA	N/a
31	−	++	2/3/2/2	+	++	3/2/2/2	−	++	1/2/1/2	N/a	N/a	N/a	N/a
32	−	++	1/2/2/3	+	++	2/2/2/2	N/a	N/a	N/a	−	++	2/2/2/2	N/a
33	−	+	0/2/2/3	−	+	1/0/0/3	−	+	0/0/0/3	−	++	1/0/0/3	N/a
34	+	++	2/3/3/0	N/a	N/a	N/a	+	++	2/2/2/0	+	++	2/2/2/0	+
35	−	++	1/3/2/2	+	++	2/1/1/2	−	+++	2/2/2/3	−	++	2/1/1/2	N/a
36	+	++	0/1/1/0	+	+++	0/1/1/0	+	++	0/2/1/2	NA	NA	NA	N/a
37	−	−	0/1/1/0	−	++	0/2/2/0	−	+	0/0/0/1	−	++	3/3/2/0	N/a
38	−	+++	0/0/0/3	−	++	0/0/0/3	N/a	N/a	N/a	N/a	N/a	N/a	N/a
39	−	+	0/0/0/3	N/a	N/a	N/a	N/a	N/a	N/a	N/a	N/a	N/a	N/a
40	−	+	0/2/2/3	−	++	2/3/2/3	N/a	N/a	N/a	N/a	N/a	N/a	+
41	−	++	0/2/2/3	+	++	0/1/1/3	N/a	N/a	N/a	−	+++	1/3/3/2	N/a
42	−	++	2/2/0/3	+	++	2/2/1/2	N/a	N/a	N/a	−	++	3/3/1/2	N/a
43	+	+++	1/3/3/2	N/a	N/a	N/a	N/a	N/a	N/a	+	+++	1/3/2/2	N/a
44	−	+	1/2/2/1	N/a	N/a	N/a	N/a	N/a	N/a	N/a	N/a	N/a	N/a
45	−	+	0/2/1/2	N/a	N/a	N/a	N/a	N/a	N/a	N/a	N/a	N/a	N/a
46	−	+	2/3/3/1	−	+	1/2/2/1	−	+	2/1/1/0	−	+++	3/3/3/1	N/a
47	+	++	2/2/2/2	N/a	N/a	N/a	N/a	N/a	N/a	N/a	N/a	N/a	N/a
48	+	++	3/3/3/0	N/a	N/a	N/a	N/a	N/a	N/a	N/a	N/a	N/a	N/a
49	+	+++	3/3/3/1	N/a	N/a	N/a	+	++	3/2/2/0	N/a	N/a	N/a	N/a
50	+	++	3/3/3/1	+	+++	3/3/3/0	+	+++	1/2/2/2	N/a	N/a	N/a	N/a
51	+	+++	3/3/3/3	N/a	N/a	N/a	N/a	N/a	N/a	N/a	N/a	N/a	N/a
52	−	−	0/3/3/1	N/a	N/a	N/a	N/a	N/a	N/a	N/a	N/a	N/a	N/a
53	+	++	2/2/1/2	N/a	N/a	N/a	N/a	N/a	N/a	N/a	N/a	N/a	N/a
54	+	+++	2/3/3/2	N/a	N/a	N/a	N/a	N/a	N/a	−	+++	1/2/2/2	N/a
55	−	+++	0/3/3/0	+	++	0/3/3/0	+	+++	1/2/2/0	+	+++	0/3/3/0	N/a
56	+	+++	2/3/3/0	N/a	N/a	N/a	N/a	N/a	N/a	N/a	N/a	N/a	N/a
57	−	−	0/0/0/3	N/a	N/a	N/a	N/a	N/a	N/a	N/a	N/a	N/a	N/a

Where two or more of the same anatomical area were available for analysis the highest scores are presented

^a^: Not available

^b^: Negri body observed (− or +)

^c^: Viral antigen immunohistochemical labelling score (− to +++)

^d^: Perivascular cuffing score/ Gliosis score/ Satellitosis and neuronophagia score/ Artefact score

### Histopathology and IHC

Serial sections were cut at 4 μm and either stained with haematoxylin and eosin (HE) for histopathological examination or labelled immunohistochemically for viral antigen as described [19].

### Histopathological (HE) analysis

**Fig. 1:**
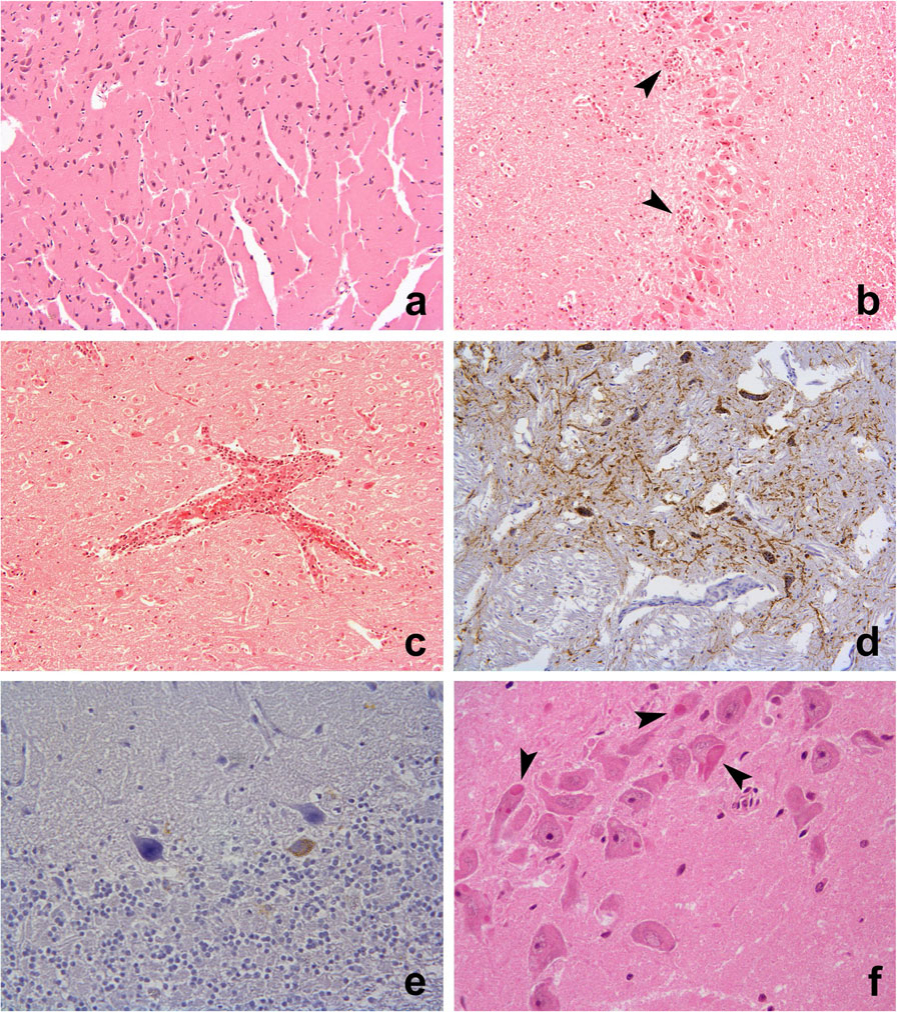
Selected histopathology and immunohistochemistry photomicrographs. **a**; Cerebrum, case 39, tissue artefact score grade 3 (HE ×10) **b**; Hippocampus, case 27, multifocal glial nodules grade 3 (HE × 10) **c**; Brainstem, case 14, perivascular cuffing grade 2 (HE ×10) **d**; Brainstem, case 29, positive grade 3 immunolabelling in grade 2 artefact tissue (IHC × 10) **e**; Cerebellum, case 3, positive grade 1 immunolabelling in grade 2 artefact tissue (IHC × 10) **f**; Hippocampus, case 12, neuronal intracytoplasmic eosinophilic viral inclusion (Negri body) in grade 2 artefact tissue (IHC × 40)

The general anatomic locations available for each case were recorded and evaluated separately as cerebrum, cerebellum, hippocampus and brain stem (including pons and medulla). The following histological features, observed in each anatomical location, were semi-quantitatively graded 0-3 by one observer (SB); (i) Perivascular cuffing (lymphocytes within the Virchow-Robins space); (ii) Gliosis (glial nodule formation); (iii) Satellitosis and neuronophagia and (iv) Artefactual degradation (freezing and autolysis) [Fig. 1]. In addition the presence or absence of Negri bodies (intracytoplasmic, 1-27 μm round, eosinophilic inclusions) [23] was recorded for each location.

### IHC analysis

Each anatomical location was semi-quantitatively graded 0-3 for the presence of specific neuronal immunolabelling by one observer (SB) [Fig. 1]. In the absence of suitable material on which to perform FAT, an IHC grade above 0 was considered a positive rabies diagnosis.

### PCR analysis (Taqman and HnRT-PCR)

One assay of each kind was performed per fresh/frozen tissue pool (13 cases) or FFPE samples (Table 1). RNA was extracted from fresh brain tissue using the TriZol™ (Invitrogen, Life technologies, Paisley, UK) method following the manufacturer’s instructions. RNA from FFPE samples was extracted from 80 μm of each individual paraffin block available from those same 13 cases using the RecoverAll™ kit (Ambion, Life technologies, Paisley, UK), and each block was tested separately one to three times. FFPE controls included a negative canine brain and two samples from a canine case that died in UK quarantine [21]. The pan-*lyssavirus* primer Jw12 was used to generate cDNA by reverse transcription. This was used to perform HnRT-PCR assays with primers Jw12 and Jw6dpl and Taqman RT-PCR assays with primers JW12 and N165-146, as previously described [16, 17, 34].

### Statistical analysis

The sensitivity of Negri body detection for the diagnosis of rabies and the presence of perivascular cuffing, gliosis and satellitosis/neuronophagia were calculated per case and by general anatomic area. The median IHC labelling and HE scores for the general anatomic areas available from positive cases were compared using a Kruskal-Wallis H test. Statistical significance was set at *p* < 0.05. Post hoc pairwise comparisons were performed using Dunn’s (1964) procedure with a Bonferroni correction for multiple comparisons; statistical significance was set at *p* < 0.0083 and only corrected *p-*values are reported. The association between artefact score and IHC labelling score was analysed using a Spearman’s rank-order correlation. A binary logistic regression was performed to ascertain the effect of artefact on the likelihood of detecting Negri bodies. PCR and IHC case results were compared but no statistical tests were required.

## Results

The general anatomic areas available for examination from each case are contained in Table 1. There was a mean of 2.3 anatomic areas per case (range 1-4) with 22 cases having a single anatomic area studied (20 positive, 2 negative), 9 with 2 anatomic areas (9 positive, 0 negative), 15 with 3 (14 positive, 1 negative) and 11 with 4 (10 positive, 1 negative). Fifty three of fifty seven cases (93%) were positive by IHC for rabies viral antigen in at least one anatomical area.

### Histopathology

Perivascular cuffing (47/53; 88.7%), glial proliferation (50/53; 94.3%) and satellitosis/neuronophagia (51/53; 96.2%) were frequently found in rabies positive cases. The mean rank of perivascular cuffing scores were not statistically significantly different, χ^2^ [3] = 6.054, *p* = .109. Glial (χ^2^(3) = 18.376, *p* = < .001) and satellitosis/neuronophagia (χ^2^(3) = 17.686, *p* = .001) scores were statistically significantly different between general anatomical areas. Post hoc analysis revealed statistically significant differences in glial scores between the cerebellum (mean rank =45.90) and hippocampus (75.65) (*p* = .050), cerebellum and cerebrum (82.04) (*p* = .001) and cerebellum and brainstem (mean rank =90.51) (*p* = < .001). Overall the cerebellum had the lowest glial score in rabies positive cases. There were no statistically significant differences between other anatomical areas. Post hoc analysis revealed statistically significant differences in satellitosis/neuronophagia scores between the cerebellum (mean rank =46.50) and cerebrum (mean rank =84.12) (*p* = .001) and cerebellum and brainstem (mean rank =88.59) (*p* = .001).

The overall sensitivity of Negri body detection in rabies positive cases was 60.4% (32/53). Negri body detection was most sensitive in the hippocampus (15/30; 50.0%) and least sensitive in the brain stem (9/34; 26.5%). Inversely, perivascular cuffing in rabies positive cases was most and least commonly observed in the brainstem (30/34; 88.2%) and hippocampus (17/30; 56.7%) respectively. Negri bodies were not found in the negative IHC cases, however in one negative case there was histopathological evidence of granulomatous meningoencephalitis. In the remaining three negative cases a cause for clinical suspicion of rabies was not determined.

### Association between artefact grade and histopathology scores

A binary logistic regression was performed to ascertain the effect of the presence of artefact on the likelihood of detecting Negri bodies. The artefact score was dichotomised as either no artefact (score 0) or artefact present (scores 1, 2 and 3). The logistic regression model was statistically significant, χ2 [1] = 8.672, (*p* = .003). The model explained 7.1% (Nagelkerke R2) of the variance in Negri body detection and correctly classified 67.7% of cases. Negri bodies were 0.34 times less likely to be detected in tissue with artefactual degeneration. Negri body detection sensitivity was almost twice as great (56.4% versus 30.8%) when there was no artefact (grade 0) compared to artefact (grade 1,2 or 3). The sensitivities of perivascular cuffing, glial proliferation and satellitosis/neuronophagia varied by less than 10% between grades when there was artefact compared to when there was none. Therefore artefact did not significantly affect HE diagnosis of encephalitis, but reduced Negri body detection.

### IHC results analysis

Specific immunolabelling was found within the perikaryon of infected neurones and within the neuropil in association with axonal processes. There was inter and intracase variability in specific patterns of labelling: including morphology, location and the number of neurones labelled, but detailed analysis was not appropriate in this series. A Kruskal-Wallis H test was conducted to determine if there were differences in rabies antigen IHC labelling scores between general anatomical areas: the brainstem (*n* = 34), cerebrum (*n* = 65), cerebellum (*n* = 24) and hippocampus (*n* = 30). IHC scores were statistically significantly different between the different general anatomic areas, χ^2^ [3] = 15.859, *p* = .001. Post hoc analysis revealed statistically significant differences in rabies antigen IHC labelling scores between the brainstem (mean rank =101.37) and hippocampus (73.88) (*p* = .045), brainstem and cerebellum (66.94) (*p* = .010) and the brainstem and cerebrum (69.41) (*p* = .001). Overall, there were significantly greater IHC scores within the brainstem as compared to other areas. The differences between cerebrum, cerebellum and hippocampus were not statistically significant.

### Association between artefact grade and IHC

A Spearman’s rank-order correlation was performed to assess the relationship between artefact score and rabies virus IHC score. There was a moderate negative correlation between increasing artefact score and IHC score, *r*_*s*_ (162) = −.260 (*p* = .001). Therefore higher artefact scores are associated with lower IHC scores. When a Spearman’s rank-order correlation was run to assess the association between artefact score and rabies virus immunoreactivity (positive/negative) there was no statistically significant correlation between increasing artefact score and overall IHC score, *r*_*s*_ (162) = −.089 (*p* = .255). Therefore case diagnosis was not affected by increasing artefact score.

### PCR assays

Of the 13 cases tested using fresh tissue, 12 were positive and one was negative for rabies. There was total agreement between IHC diagnosis (positive/negative) and the fresh tissue PCR test results. Despite multiple attempts, HnRT-PCR and Taqman RT-PCR assays were negative in the matching sample sets using FFPE tissue. Taqman RT-PCR, but not HnRT-PCR, produced positive results in the two FFPE samples used as positive technique control.

## Discussion

The purpose of this study was to assess the distribution of rabies viral antigen and inflammatory changes within the general anatomical areas of the brain alongside an evaluation of the effect of artefact and suitability of FFPE tissue for molecular diagnosis of rabies virus. In this large canine case series Negri bodies were present in 60% (32 of 53) of the positive cases, which is similar to a relatively recent report [1]. Similarly, as previously reported in canine brain, Negri bodies were most often detected in the hippocampus [3, 23]. There have been reports of rare Negri body-like inclusions occurring in rabies negative canine brain, however in this series inclusions were not observed in IHC negative cases [26, 28].

The observation of Negri bodies was associated with lower scores of autolysis and freeze thaw artefact. Therefore, whilst of some value if no other method is available; the sensitivity of Negri body detection is such that other complementary tests must be employed to confirm or refute a diagnosis particularly in autolytic specimens.

The anatomical distribution of inflammation observed with HE staining is relevant to sampling site identification, pathogenesis and immunological studies. The pons and medulla have previously been identified as the site of greatest inflammation in a murine model and the brainstem has been described as the site of most intense inflammation in naturally infected paralytic forms of canine rabies [18, 19, 30]. In this series, rabies positive cases also exhibited perivascular cuffing, most frequently in the brain stem and least often within the hippocampus, where Negri bodies were more frequently detected. Negri bodies were least often identified in the brainstem; potentially this is because the inflammatory response is destructive and may obscure Negri body observation. The relationship between inflammation and paralytic or furious clinical presention has previously been described [30] but was not possible in this study because the clinical information was unavailable.

The cerebellar glial score was significantly lower than other areas and satellitosis/neuronophagia score was significantly greater in the cerebrum and brainstem. The relative increase in inflammation within the brainstem, as compared to other structures, may be related to the natural time course of infection, whereby it is infected via retrograde transport of virus antigen from a distal site of inoculation (eg bite wound) before the cerebellum/hippocampus. Therefore, earlier local infection results in increased upregulation of inflammatory cytokines and lymphocyte perivascular cuffing, resulting in more severe leukocyte recruitment and inflammation. This is supported by previous work where mice were inoculated in the foot pad with *Lyssavirus*: increased immunolabelling of CCL2, CCL5 and CXCL10 was identified in caudal brain regions, particularly the medulla and pons associated with more severe inflammatory changes [18].

The IHC labelling score for rabies viral antigen was significantly greater within the brainstem. This is in agreement with previous investigations using fresh tissue with FAT and IHC labelling [5, 30] although contrary to two much smaller canine series [1, 31]. Viral entry into the brain is expected via the brainstem because natural canine infections occur from bites, either directly from a facial injury or via the spinal cord when limbs are affected. Other authors have suggested that the viral location in cholinergic rich areas of the brain is an intrinsic property of the rabies virus [32]. Subsequent further local spread to higher structures such as the cerebrum, hippocampus and cerebellum would then logically follow.

Viral antigen labelling score is greater within the brain stem, while Negri bodies are most frequently observed within the hippocampus. The reasons for this are unclear but this change may reflect the local destructive effects of inflammation within the brainstem (as previously discussed) that obscures Negri body visualisation, or simply the convenience of observing viral inclusions within large Purkinje neurones that are present in high numbers within the dentate gyrus. An undefined predisposition of Purkinje neurones to accumulate viral antigen, without an accompanying florid inflammatory response, cannot be excluded; but it would seem more likely that inflammation would follow viral antigen accumulation. It is therefore possible that the clinical effects of brain stem inflammation have supervened before hippocampal lesions have had time to progress and therefore the observed differences in the hippocampus and brain stem areas reflects the natural course of infection.

Less invasive necropsy techniques such as sampling the brain using a straw via the occipital route [5], has been validated for use in the field where full brain exposure and removal of the encephalon is impractical and carries an associated operator risk [36].

Artefactual degeneration was associated with reduced immunoreactivity, but it did not affect the overall positive or negative diagnosis. Formalin fixed tissue can be stored without undergoing further bacterial and autolytic degeneration prior to accessing laboratory facilities. Excessive cross-linking from prolonged formalin fixation may affect antigen retrieval, but this was not significant in the studied series [29]. Therefore IHC labelling of FFPE tissue offers an ideal method for rabies diagnosis when artefactual degeneration of fresh brain tissue is inevitable or where fresh tissue is not available from suspect cases.

Although FAT is the OIE prescribed tool for rabies diagnosis in animals there are reported weaknesses of the FAT: autolysis and bacterial contamination are known to substantially reduce sensitivity [1, 2, 12] and formalin fixation may make a sample unsuitable for routine testing [29, 36]. IHC labelling of brain tissue has previously been reported to be as sensitive as FAT, with possibly increased sensitivity in autolysed tissue [1, 36]. IHC labelling has been used as an additional confirmatory test in clinical cases [21, 35] but is not a routine diagnostic technique endorsed by the OIE [36]. The time taken to acquire a result, as compared to FAT, is relatively slow [29] alongside the potential for false positive or negative results as a consequence of relying on poorly trained staff [36].

Rabies viral RNA extraction from available fresh and FFPE tissue was undertaken with subsequent successful HnRT-PCR and Taqman RT-PCR testing of fresh tissue. The results from this were in full agreement with the IHC diagnoses, providing evidence of concordance between the two methods in the absence of FAT testing. The artefact score of the tested samples encompassed the full range of observed preservation (score 0 to 3). PCR has previously been reported as a very reliable method for rabies diagnosis despite sample deterioration, which appears to have been the case in this study [8, 25].

The PCR tests undertaken using FFPE tissues from Sri Lanka were negative. The primers used (JW12-JW6dpl) for HnRT-PCR have an analytical sensitivity of 0.01 FFD_50_ [2] but the amplified fragment is relatively large at 606 base pairs [21]. Samples that have been fixed in formalin, and particularly prolonged fixation at warm temperatures, are subject to RNA fragmentation: this may be from formalin induced cross linking, RNAse activation or both [4, 6, 22]. This fragmentation means that retrieval of intact large sequences above approximately 200-300 bp from FFPE tissue has been shown to be unlikely to succeed [22]. This may explain the negative results using HnRT-PCR and implies that this technique is unlikely to ever succeed on formalin fixed tissue unless alternative extraction methods, which reverse formalin induced cross linking, are developed.

The Taqman RT-PCR (JW12 and N165-146) is reported to be 200 fold more sensitive than the HnRT-PCR, with a much shorter amplicon of approximately 100 base pairs [16, 34]. This assay is therefore more likely to provide positive results from infected FFPE tissue. The Sri Lankan FFPE test material was also negative in this series however APHA processed control material was positive. The reasons for this may potentially lie in the age of the tissue, method of storage and time from processing to analysis. The Sri Lankan test material was up to 2 years older than the control tissue, subject to an unknown length of fixation and type of storage, whereas APHA material had been fixed for 7 days, processed to wax and stored in optimal humidity and temperature controlled conditions. The effect of wax embedding on RNA degradation is also unknown. Previously, real time PCR has been used to amplify a 126 bp sequence successfully for typing of a specific African *Lyssavirus* (isolate 864/09) from formalin fixed (but not paraffin embedded) tissue [7]. This might mean that results using formalin fixed, but unprocessed material may be more likely to be successful. This would reflect commonly encountered diagnostic field samples.

In this series, detailed anatomical and cellular analysis was not appropriate because of sample degeneration; however this reflects the nature of ‘field’ cases. Additionally every anatomical region was not available for each case. The cerebrum was over represented, possibly because the most dorsal structure is large and therefore relatively easy to sample when the entire brain is exposed at necropsy or if the prosector is unfamiliar with rabies diagnosis; for similar reasons the brain stem is under represented.

## Conclusions

HE examination is essential to define the differential diagnoses of behaviour modifying conditions in rabies virus negative cases and for future analysis of viral pathogenesis, but it is unreliable as the sole method for rabies diagnosis, particularly where artefactual change has occurred. Formalin fixation and paraffin embedding does not prevent detection of rabies virus via IHC labelling even where artefactual degeneration has occurred. This could represent a pragmatic secondary assay for rabies diagnosis in the field because formalin fixation is a convenient and economical way to prevent sample degeneration. The brain stem was shown to be the site with most viral immunoreactivity, supporting recommended sampling protocols in favour of improved necropsy safety in the field. The use of HnRT-PCR for testing of FFPE tissue is unlikely to be successful but Taqman RT-PCR may offer a better chance for success.

## Abbreviations

FAT: Fluorescent antibody test
FFPE: Formalin fixed paraffin embedded
HE: Haematoxylin and eosin
HnRT-PCR: Hemi nested reverse transcription polymerase chain reaction
IHC: Immunohistochemical
PCR: Polymerase chain reaction
RT-PCR: Reverse transcription polymerase chain reaction
